# Metagenomic Insights into the Bioaerosols in the Indoor and Outdoor Environments of Childcare Facilities

**DOI:** 10.1371/journal.pone.0126960

**Published:** 2015-05-28

**Authors:** Su-Kyoung Shin, Jinman Kim, Sung-min Ha, Hyun-Seok Oh, Jongsik Chun, Jongryeul Sohn, Hana Yi

**Affiliations:** 1 BK21PLUS Program in Embodiment: Health-Society Interaction, Department of Public Health Sciences, Graduate School, Korea University, Seoul, Republic of Korea; 2 School of Biological Sciences & Interdisciplinary Program in Bioinformatics, Seoul National University, Seoul, Republic of Korea; 3 School of Biosystem and Biomedical Science, Korea University, Seoul, Republic of Korea; California Department of Public Health, UNITED STATES

## Abstract

Airborne microorganisms have significant effects on human health, and children are more vulnerable to pathogens and allergens than adults. However, little is known about the microbial communities in the air of childcare facilities. Here, we analyzed the bacterial and fungal communities in 50 air samples collected from five daycare centers and five elementary schools located in Seoul, Korea using culture-independent high-throughput pyrosequencing. The microbial communities contained a wide variety of taxa not previously identified in child daycare centers and schools. Moreover, the dominant species differed from those reported in previous studies using culture-dependent methods. The well-known fungi detected in previous culture-based studies (*Alternaria*, *Aspergillus*, *Penicillium*, and *Cladosporium*) represented less than 12% of the total sequence reads. The composition of the fungal and bacterial communities in the indoor air differed greatly with regard to the source of the microorganisms. The bacterial community in the indoor air appeared to contain diverse bacteria associated with both humans and the outside environment. In contrast, the fungal community was largely derived from the surrounding outdoor environment and not from human activity. The profile of the microorganisms in bioaerosols identified in this study provides the fundamental knowledge needed to develop public health policies regarding the monitoring and management of indoor air quality.

## Introduction

There are numerous microorganisms in the air we breathe; the number of bacterial cells and fungal spores present in air is estimated to be 10^4^·m^-3^ [1,2] and ~10^3^–10^4^·m^-3^ [3–5], respectively. Although the atmosphere is an extreme environment for microorganisms because of high levels of solar radiation, low moisture, and low nutrient content, many of these airborne microbes are metabolically active [6,7]. Both the metabolically active and inactive airborne microbes have clinically important effects on human health. For example, exposure to airborne fungal allergens such as hyphae, spores, and fungal fragments can cause asthma, rhinitis, atopic dermatitis, and allergic diseases [8–12]. Young children have higher susceptibility to bioaerosols than adults; hence, they are more vulnerable than adults to the diseases caused by microorganisms in bioaerosols. In addition, they breathe more air than adults (per kilogram of body weight), which may result in a higher daily intake of the pathogens or allergens present in air [13,14]. In Korea, children aged 4–9 years generally attend daycare centers or elementary schools for one quarter of each day. Therefore, determining the components of the bioaerosols in these spaces is important for improving the public health of children.

For a long time, culture-based microbiological methods have been used to investigate airborne microbial communities, and these methods have enabled direct observation of airborne bacteria and fungi. Culture-based studies have shown that most airborne bacteria are typically gram-positive bacteria belonging to the genera *Staphylococcus*, *Corynebacterium*, *Bacillus*, and *Micrococcus* [15–21]. However, some gram-negative bacteria, such as *Acinetobacter*, *Moraxella*, *Pantoea*, and *Pseudomonas* were also isolated at lower frequencies [19]. The dominant airborne fungi detected belonged to the genera *Aspergillus*, *Penicillium*, *Cladosporium*, and *Alternaria* [15,22,23]. However, culture-based studies can only provide an estimate of the true diversity of the microbial communities in air because most (>99%) microorganisms in the environment are recalcitrant to culturing [24,25]. In fact, the composition of the airborne microbial communities determined by culture-dependent and culture-independent 16S rRNA gene sequencing differ considerably [26,27].

Since the 1990s, new molecular taxonomic techniques for fungi have been introduced, and DNA sequence databases have been developed to facilitate fungal identification. These molecular identification tools have provided new insights into fungal ecology. In recent culture-independent studies of airborne fungi, the ITS region was chosen as a target molecular marker [28–30]. Yamamoto *et al*. [31] identified 558 different genera in outdoor air samples by analyzing the entire ITS region. Fröhlich-Nowoisky *et al*. [32] used DNA sequence analysis to efficiently detect and unambiguously characterize fungi in atmospheric aerosol samples. The ITS region is currently the most phylogenetically informative sequence for fungal identification, especially at lower taxonomic levels.

In recent years, metagenomic approaches have provided deeper insights into airborne microbial diversity in different environments, such as urban environments [31,33–36], subway systems [37,38], museums [39], buildings [40,41], and air particles [32,42]. However, the bioaerosols in children’s daytime spaces have not been extensively examined in metagenomic studies. Thus, we investigated the diversity of microorganisms present in the air of education facilities for children in Seoul, Korea using 454 pyrosequencing. The bacterial and fungal populations in 50 indoor and outdoor air samples from five daycare centers and five elementary schools were analyzed. The purpose of this study was (1) to characterize the microbial composition of the air at locations frequented by children, and (2) to examine the association between indoor and outdoor bioaerosols. The results of this study will improve our understanding of airborne microorganisms and provide a rationale for developing public health policies regarding the monitoring and management of indoor air quality.

## Materials and Methods

### Sample collection

Indoor and outdoor air samples were collected at five daycare centers (sites A to E) and five elementary schools (sites F to J) located in Seoul, Korea from August 19 to October 24, 2013. The presidents of the elementary schools and the directors of the daycare centers were provided official documents from the Research and Business Foundation of Korea University requesting their cooperation, and they gave permission to conduct the study at each site. The results of this study were communicated to the daycare centers and elementary schools. Information about the investigated daycare centers and schools is shown in Table 1. The facilities were selected because the buildings are widely distributed in Seoul and are nearly equidistant to each other. The evaluated daycare centers and schools varied in building construction, size, surrounding environment, and the number of children served. Ventilation was provided naturally through windows. The children performed their normal activities during the measurements. Temperature and relative humidity were monitored during the sampling periods. The microbiological sampling cycle was of 10 h (from 8 am to 6 pm). To obtain duplicate or triplicate samples from the same site, the sampling sessions were conducted for 2 subsequent days (24 h periods) for the daycare centers and 3 subsequent days for the elementary schools. Samples were obtained using an air sampler equipped with Millipore cassettes (47 mm diameter) with sterile mixed cellulose ester filters (47 mm; 0.45 μm; Millipore). Although microsized (<0.45 μm) bacteria could pass though the 0.45 μm pores, we chose this filter based on the results of previous studies [36,43,44] and initial testing, in which we verified an increase in bacterial mass by agglomeration of abiotic or biotic particles on the filter membrane during sampling. The air sampler was operated at an airflow rate of 24 L/min, which was maintained by a vacuum pump (BMW-200; Total Eng) and verified with a calibrator (TSI-4045 Mass Flowmeter; TSI) during each 24-h measurement. For indoor air measurement, the air sampler cassette was fixed on a tripod 1.5 m above the floor in the rear of the classroom. The pumps for the indoor air samplers were placed inside noise-insulated enclosures to reduce the noise exposure of the occupants. The outdoor air measurements were obtained approximately 1.5 m above the ground outside the building, avoiding areas traversed by people. Before sampling, the empty sampling cassettes were sterilized in an autoclave and then dried in a drying oven. The samples were transported directly to the laboratory under chilled conditions (4°C). Then, the filters were placed in sterile plastic tubes and frozen at -20°C until DNA extraction.

**Table 1:** Characteristics of the investigated child daycare centers and elementary schools.

	Site	Sampling date	Location	Age of occupants	Mean temp. (°C)		Mean humidity (%)	
					Indoor	Outdoor	Indoor	Outdoor
Daycare centers	A	2013.08.19~20	127°1´11.8˝E,37°37´39.6˝N	5	26.7	28.9	56.8	57.6
	B	2013.08.21~22	127°1´40.5˝E,37°39´32.9˝N	5	28.8	29.4	47.7	53.3
	C	2013.08.26~27	127°0´50.9˝E,37°39´15.1˝N	5	30.2	29.5	45.2	46.0
	D	2013.08.28~29	127°7´13.7˝E,37°32´7.3˝N	5	29.6	28.3	56.5	66.4
	E	2013.09.30~01	127°2´36.1˝E,37°40´43.0˝N	5	24.0	24.1	63.3	60.0
Elementary schools	F	2013.09.02~04	127°3´58.1˝E,37°40´2.4˝N	8	28.4	24.3	45.9	49.5
	G	2013.09.09~11	127°2´6.5˝E,37°35´41.2˝N	8	26.6	24.1	59.1	70.6
	H	2013.09.24~26	127°0´54.7˝E,37°34´2.2˝N	8	26.7	23.7	45.6	52.4
	I	2013.10.14~16	126°52´55.4˝E,37°34´47.3˝N	9	26.0	16.9	38.1	59.8
	J	2013.10.21~23	126°57´6.8˝E,37°33´32.7˝N	8	23.1	20.5	37.9	41.5

### DNA extraction, PCR, and pyrosequencing

DNA was extracted directly from each air sampling filter using a commercial soil DNA isolation kit (MP Biomedicals). The extracted DNA was amplified using primers targeting the V1–V3 regions of the prokaryotic 16S rRNA gene as previously described [45]. The primers used for bacteria were V1-9F (5′-CCTATCCCCTGTGTGCCTTGGCAGTC-TCAG-AC-GAGTTTGATCMTGGCTCAG-3′ [the underlined sequence is the gene-specific region]) and V3-541R (5′-CCATCTCATCCCTGCGTGTCTCCGAC-TCAG-barcode-AC-WTTACCGCGGCTGCTGG-3′) [45]. The ITS regions of the fungal rRNA operon were amplified using fusion primers ITS-3F (5′-CCTATCCCCTGTGTGCCTTGGCAGTC-TCAG-CA-CATCGATGAAGAACGCAGC-3′) and ITS-4R (5′-CCATCTCATCCCTGCGTGTCTCCGAC-TCAG-barcode-GC-TCCTCCGCTTATTGATATGC-3′). The fusion primers contained 454-specific adapters, keys, linkers, barcodes, and universal fungal ITS priming sequences [46]. The barcode and primer sequences are available at http://www.ezbiocloud.net/resource/M1001. The PCR for both bacteria and fungi was performed under the following cycling conditions: an initial denaturation step at 94°C for 5 min, followed by 10 cycles of denaturation at 94°C for 30 s, annealing at 60°C to 55°C (with a touchdown program) for 45 s, and elongation at 72°C for 90 s. This was followed by an additional 20 cycles of denaturation at 94°C for 30 s, annealing at 55°C for 45 s, and elongation at 72°C for 90 s, and a final elongation step at 72°C for 5 min. The sizes of the amplicons were 500 bp–700 bp for bacteria and 600 bp–800 bp for fungi, respectively. The amplified products were purified using resin columns (Qiagen), and 1 μg of the PCR product from each sample was mixed and purified using the AMPure bead kit (Agencourt Bioscience). The DNA was sequenced unidirectionally from universal primers (518R for bacteria and ITS-4R for fungi) at Chunlab, Inc. with a Roche/454 GS Junior system according to the manufacturer’s instructions. The sequencing data from this study were deposited in the Short Read Archive under accession number SRP043178 (Bioproject accession number PRJNA252641).

### Processing of sequencing data

The pyrosequencing data for the 16S rRNA gene sequences was processed through Java-based multi-step bioinformatics pipelines as described elsewhere [45,47–49]. The unidirectional sequencing reads from different samples were separated by their unique barcodes. To filter low quality sequences, reads <300 bp or with an average quality score <25 were omitted. Then the barcode, linker, and PCR primer sequences were removed from both sides of the reads using pairwise sequence alignment and the hmm-search program in HMMER 3.0 [50]. The trimmed sequencing reads were assembled into sets of highly similar sequences using a TBC clustering algorithm with a 97% cutoff [51]. While clustering, homopolymeric errors were ignored by allowing a mismatch error of up to 2 bp, which was based on the error rate of 454 sequencing (0.5%). Representative sequences in clusters of trimmed sequences were chosen for identification. Singletons were considered as individual OTUs. The representative sequences and singletons were assigned to taxonomic positions according to the highest pairwise similarity among the top five BLASTN hits against the EzTaxon-e database [52]. Sequences that showed no match in a BLASTN search (expectation value of >e^−5^) against the EzTaxon-e database were considered to be non-target sequences and were ignored. To calculate the nucleotide sequence similarity between the query and the candidate species, Myers and Miller global pairwise alignment [53] was used along with CLUSTAL [54]. Chimeric sequences were detected by UCHIME [55] and were eliminated from further processing.

To analyze the fungal sequences, the sequencing reads were processed by Fungal ITS Extractor [56], which uses a profile hidden Markov model to obtain pure ITS sequences. The extracted ITS sequences were filtered and denoised using the same pipeline used for the bacterial sequence analysis, except that the clustering was not performed. Individual reads were subjected to a BLASTN search against the UNITE (https://unite.ut.ee/) and EzFungi (http://www.ezbiocloud.net/ezfungi) databases. Sequences that showed no match in the BLASTN search (expectation value of >e^−5^) were considered to be non-target sequences and were ignored.

### Statistical analyses

To avoid potential bias caused by different sequencing depths, samples with more than 3,000 reads were rarefied to a depth of 3,000 reads (2,000 reads for fungal sequences) for subsequent analysis. The species richness (rarefaction curves) and diversity indices (OTUs richness, Chao1 richness [57], and Shannon diversity [58,59]) were calculated using the rRNA Database Project’s pyrosequencing pipeline (http://pyro.cme.msu.edu/) using the CLcommunity program (http://www.chunlab.com/software_clcommunity_about). The diversity measures were calculated by using a TBC clustering algorithm, and the cutoff value for assigning a sequence to a species-level OTU was ≥97% similarity. Source tracking using CLcommunity software was performed to determine the proportion of the communities that originated from human sources. The percentage of the sequencing reads that were shared between a sample community and a reference human microbiome was calculated. The overall phylogenetic distance between each pair of communities was estimated using Fast UniFrac analysis (29) in the CLcommunity program. In brief, sequences were first identified at the species level using a similarity-based identification method with the EzTaxon-e or EzFungi database. The taxonomic assignments were applied to a reference phylogenetic tree backbone constructed from the EzTaxon-e or EzFungi taxonomic structure. Sequences that could not be assigned to known taxa were considered to belong to different species. Using the reference tree with species abundance values, a weighted Fast UniFrac distance was calculated for the samples. The resultant distance matrix was then used to generate an ordination diagram using principal coordinate analysis (PCoA) in the R program (http://www.r-project.org/). For fungal communities, Bray-Curtis dissimilarity-based PCA analysis was also performed using the genera abundance table.

To compare the microbial community structures based on categorical metadata, samples were pooled into binds (daycare/elementary school or indoor/outdoor), and statistical significance tests were performed using the R program. Differences in diversity indices, depending on categorical metadata, were evaluated by ANOSIM using the Wilcoxon *t*-test. The significance of differences in the microbial profiles (the PCoA vectors from UniFrac distance analysis) according to categorical metadata was determined using Wilcoxon *t*-test on the x- and y-coordinates. The difference in species abundance, depending on categorical metadata, was determined using Hotelling’s *t*-test, and multiple testing problems were adjusted with false discovery rate correction [60]. The correlation between environmental variables (temperature or humidity) and microbial community was evaluated using linear regression analysis.

## Results

### Overview of the microbial diversity in aerosols

Fifty samples were collected from the indoor and outdoor air of five daycare centers (sites A to E) and five elementary schools (sites F to J) (Table 1). The bacterial and fungal communities in all the samples were successfully characterized (S1 Table). Pyrosequencing of bacterial 16S rRNA gene amplicons resulted in 254,771 valid reads (average length, 458 nt) for the 50 air samples (average, 5,095 reads/sample). We observed an average of 1,440 bacterial operational taxonomic units (OTUs) for each sample (range, 144–5,027). The Chao1 estimator of species richness ranged from 173 to 6,599 (average, 1,788). The observed OTU richness of the indoor and outdoor samples did not differ significantly (Wilcoxon *t*-test). Bacterial community diversity, as estimated by the Shannon diversity index, between indoors and outdoors or between daycare centers and elementary schools did not differ significantly.

Pyrosequencing of fungal ITS amplicons resulted in 195,092 valid reads (average length, 339 nt) for the 50 air samples (average, 3,902 reads/sample; S1 Table). Fungal richness and diversity were estimated based on the OTUs with 97% nucleotide sequence similarity. The number of OTUs ranged from 149 to 697 for each sample (average, 390). The Chao1 estimator of species richness ranged from 292 to 1,155 depending on the sample (average, 629), indicating that approximately 1.61-fold more fungal OTUs may have been present in the collected air samples than were actually observed in this study. The observed OTU richness and the fungal community diversity, as estimated by Shannon diversity index, did not differ between the indoors and outdoors or between the daycare centers and elementary schools.

In the rarefaction analyses of bacterial or fungal reads, although the gradients of the collector’s curves decreased with increasing numbers of sequences, the number of OTUs still increased even at the highest number of sequences samples, indicating the high bacterial diversity in tested samples (data not shown).

### Community structure dynamics

**Fig 1:**
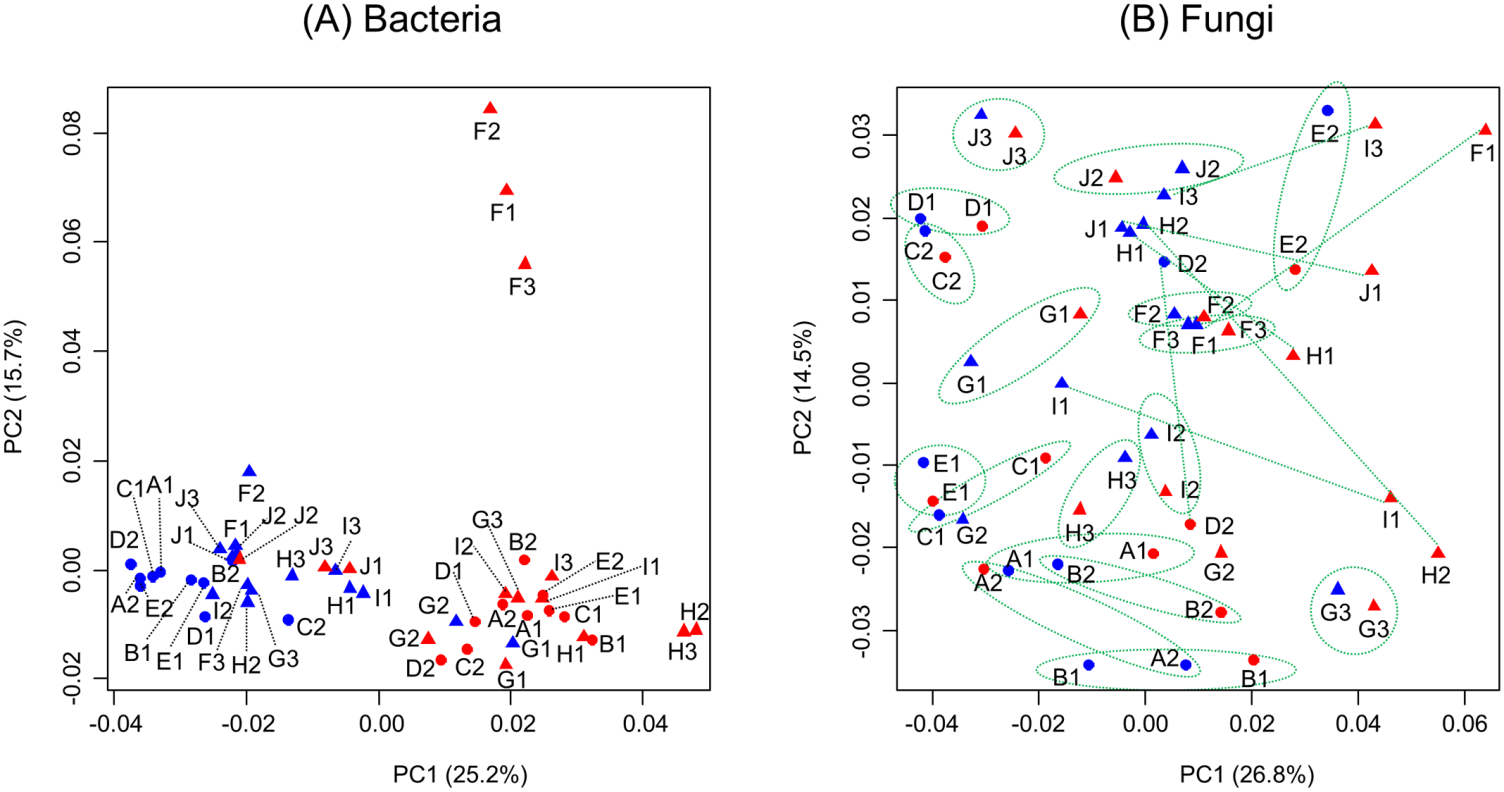
Principal coordinate analysis (PCoA) of the (A) bacterial and (B) fungal communities in childcare facilities. Fifty samples were collected from the indoor (blue symbols) and outdoor (red symbols) air of five daycare centers (sites A–E, circles) and five elementary schools (sites F–J, triangles). Differences in the composition of the microbial communities were quantified using the weighted UniFrac distance metric. The green dotted lines and circles indicate pairs of indoor and outdoor samples from the same sampling site. The distinctive bacterial community structures in the indoor and outdoor air were supported by the p-values determined by Wilcoxon *t*-test.

The bacterial composition of the indoor samples was generally different from that of the outdoor samples. In the Fast-UniFrac distance-based PCoA analysis, the indoor and outdoor samples formed distinct clusters (Fig 1A; Wilcoxon *t*-test, p < 0.0000 and p = 0.0022 for the x-coordinates and y-coordinates, respectively). A few exceptional cases were also observed, such as at sites J, G, and F. Although the J-Out samples were from outdoor air, they contained a large proportion of human-associated bacteria, including species belonging to the genera *Micrococcus*, *Paracoccus*, and *Staphylococcus*. The G-In samples (from indoor air) contained a high proportion of *Actinobacteria*, which are usually abundant in outdoor air. Significant differences between the F-out samples and other outdoor samples were driven by the idiosyncratic abundance of bacteria belonging to the genera *Streptomyces*, *Pseudonocardia*, and *Nocardiopsis*.

In addition, differences were also observed between the indoor bacterial communities in daycare centers and elementary schools (p = 0.0001 [x-coordinates] and p = 0.8918 [y-coordinates]). The bacterial communities outside of daycare centers and elementary schools did not differ.

In contrast to the bacterial community, the fungal community composition in the indoor and outdoor air did not differ. This was evident in both the EzFungi-based UniFrac analysis and the UNITE-based Bray-Curtis dissimilarity analysis (S1 Fig). The air samples tended to cluster according to sampling site, regardless of whether they were of indoor or outdoor origin (Fig 1B). For example, the indoor and outdoor samples from the J3 site were very similar (upper left side of the ordination graph). This result indicates that the fungal communities of the indoor air reflected those of the outdoor air; therefore, human activity had little influence on the indoor fungal composition.

The effect of temperature and relative humidity on the abundance of specific microbial taxa was evaluated using regression analyses; however, no direct correlation was observed. In addition, the overall bacterial community structure (PCoA coordinates) was not linked to environmental variables. This may be because the duration of sampling was as long as 10 h, and the samplings were restricted to the same city over a short period of time.

### Bacterial community composition

In the outdoor and indoor air, 38 and 32 bacterial phyla were detected, respectively. Most sequences (60–96.3%) were assigned to 3 dominant phyla, namely, *Actinobacteria* (37% indoor, 35% outdoor), *Proteobacteria* (34% indoor, 33% outdoor), and *Firmicutes* (18% indoor, 15% outdoor). The phyla *Cyanobacteria*, *Bacteroidetes*, *Chloroflexi*, *Acidobacteria*, and *Deinococcus-Thermus* made minor contributions (<5% on average) to the overall population. Other phyla contributed less than 1% (on average) of the total sequences. Only a few sequences fell into candidate phyla (e.g., TM7, BRC1, and OD1; <1% of each sample).

**Fig 2:**
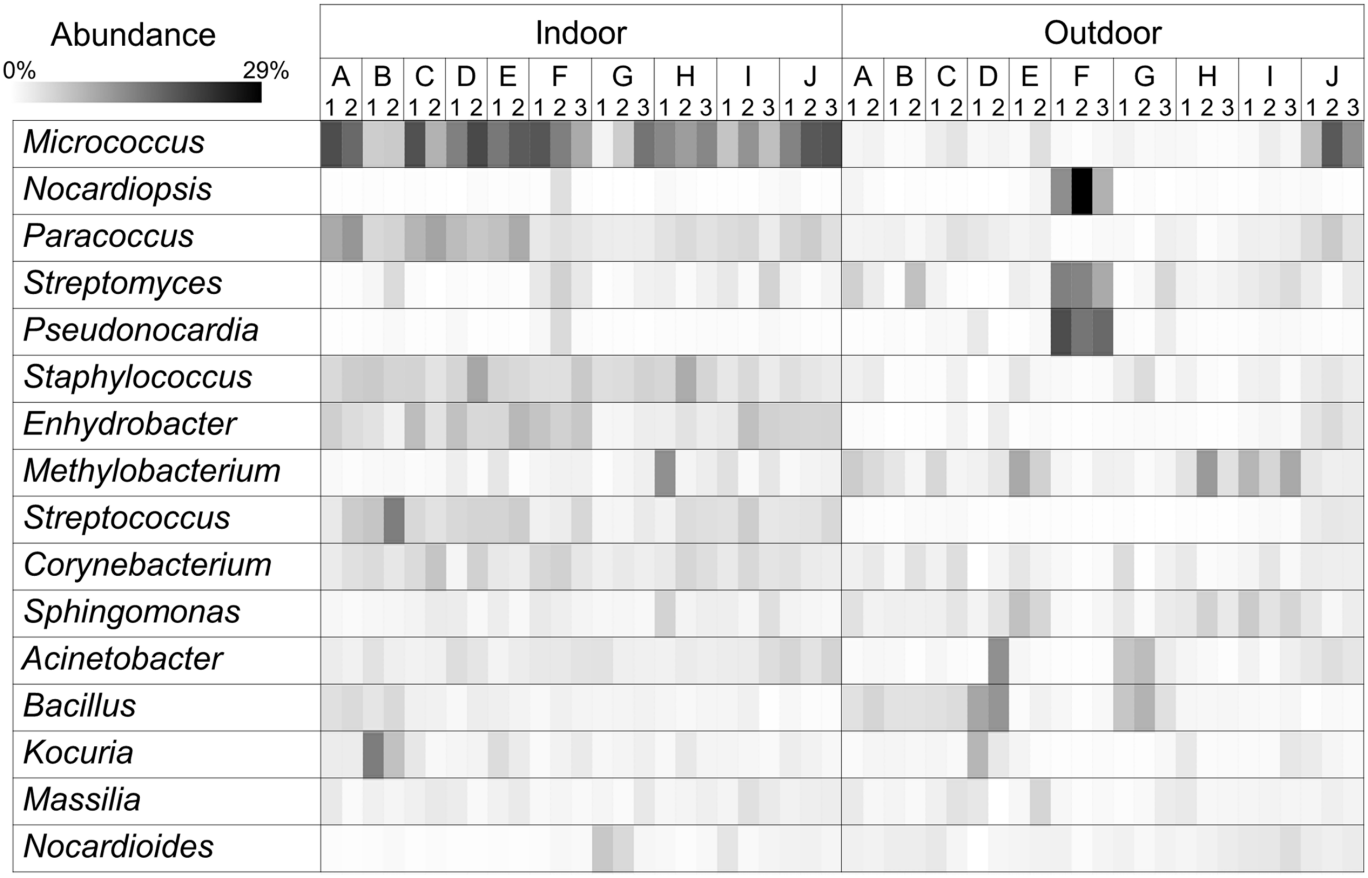
Relative abundance of bacteria identified at the genus level in indoor and outdoor air. The sampling sites and replicates are indicated as letters and numbers, respectively, shown in columns. The taxonomic groups that had an average abundance >1.2% are shown.

At the generic level, 2,537 bacterial genera were detected in the indoor and outdoor samples of this study. The distributions of the most prominent bacterial genera (>1.2% of the total bacteria) in the 50 air samples are shown in Fig 2. The bacterial genera at >1.2% abundance accounted for 50.0% and 41.3% of the bacteria in the indoor and outdoor samples, respectively. Of the bacterial genera identified in the indoor air samples, the genus *Micrococcus* was the most abundant (13.2%), followed by *Paracoccus* (5.2%), *Staphylococcus* (4.6%), and *Enhydrobacter* (4.3%; S2 Table).

The composition of the dominant bacteria in the indoor air clearly differed from that in the outdoor air. The indoor air samples contained a greater number of well-documented human-associated bacteria than the outdoor air; *Micrococcus* (13.72% indoor, 3.3% outdoor; p < 0.0001), *Staphylococcus* (4.2% indoor, 1.3% outdoor; p < 0.0001), *Streptococcus* (4.2% indoor, 0.2% outdoor; p < 0.0001), *Corynebacterium* (3.3% indoor, 1.7% outdoor; p = 0.0008), and *Propionibacterium* (1% indoor, 0.3% outdoor; p = 0.0006; S2 Fig).

In contrast, in the outdoor samples, several genera commonly found in soil and water were generally abundant, *Methylobacterium* (3.9%), *Streptomyces* (3.5%), *Pseudonocardia* (2.8%), *Sphingomonas* (2.6%), and *Bacillus* (2.5%; S2 Table). The composition of the abundant bacterial genera in the outdoor air samples was highly variable. For example, the most common genus varied between the outdoor samples: *Bacillus* (12.1%) in D2-Out, *Sphingomonas* (7.2%) in E1-Out, *Pseudonocardia* (20.83%) in F1-Out, and *Methylobacterium* (12.2%) in H1-Out.

Because the indoor air samples from daycare centers and elementary schools also differentiated on the PCoA plot, the abundance of the dominant bacteria was compared. The genera *Paracoccus* (8.1% daycare centers, 3.4% elementary schools; p = 0.0001), *Streptococcus* (5.7%, 2.9%; p = 0.0313), and *Bacillus* (2.2%, 0.95%; p = 0.0049) were more abundant in daycare centers than in elementary schools.

### Fungal community composition

**Fig 3:**
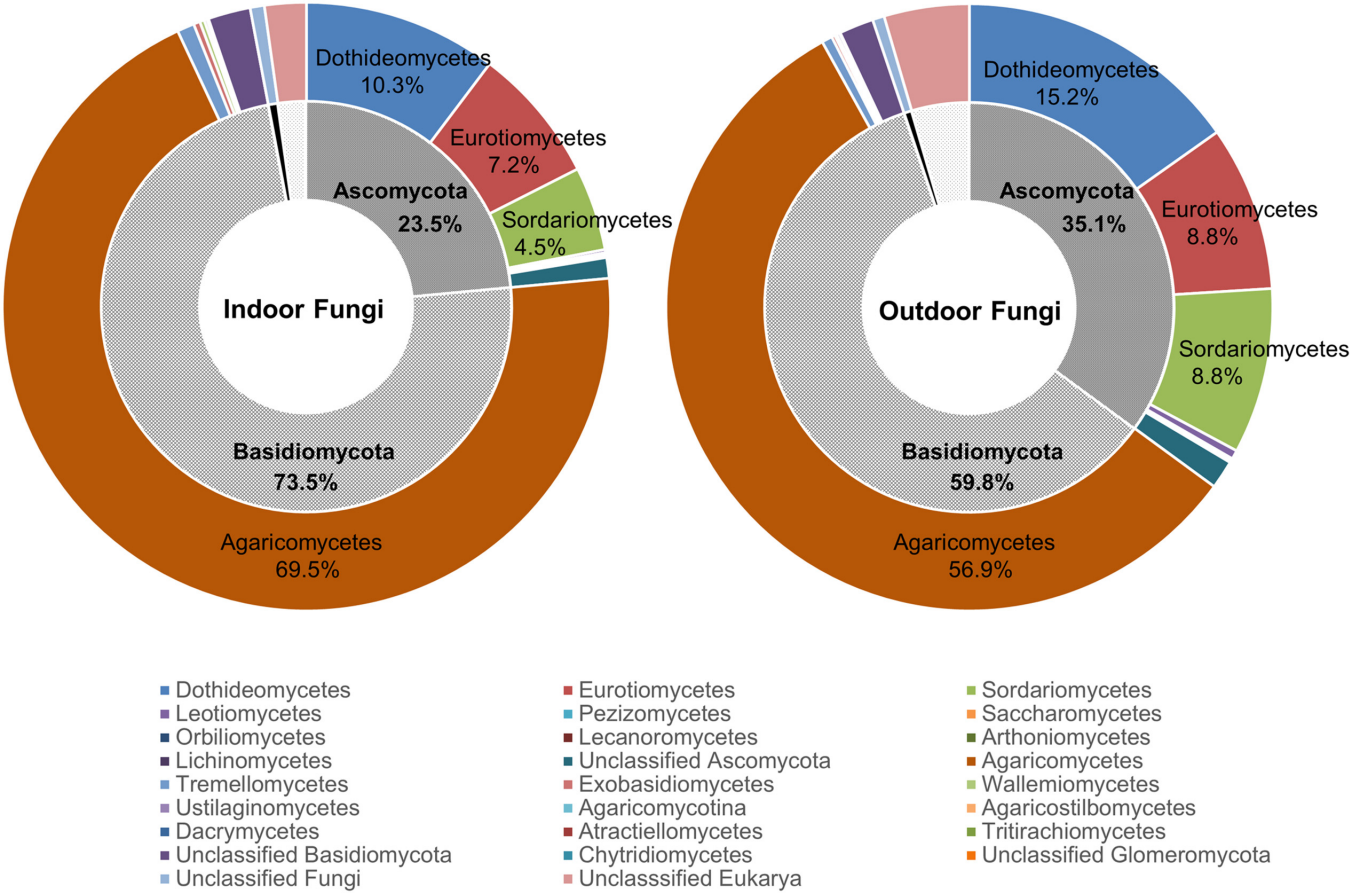
Fungi in indoor and outdoor air identified at the phylum and class levels. The inner circles indicate the composition of fungal reads at the phylum level, and the outer circles indicate the composition of the fungal community at the class level.

Based on the EzFungi database, most ITS reads belonged to *Basidiomycota* (73.5% indoor, 59.8% outdoor) and *Ascomycota* (23.5% indoor, 35.1% outdoor; Fig 3). A very low proportion of *Chytridiomycota* (0% indoor, 0.001% outdoor) and *Glomeromycota* (0.001% indoor, 0% outdoor) was detected. The rest of the identified sequences belonged to unclassified fungi that could not be attributed to a phylum.

The airborne *Ascomycota* encompassed three major fungal classes, namely *Dothideomycetes* (10.3% indoor, 15.2% outdoor), *Eurotiomycetes* (7.2% indoor, 8.8% outdoor), and *Sordariomycetes* (4.5% indoor, 8.8% outdoor; Fig 3). Depending on the sample, 0.2% to 57.7% of the reads could not be attributed to a class and were regarded as unidentified sequences. *Dothideomycetes*, which includes genera associated with allergenic fungi such as *Alternaria*, *Epicoccum*, *Curvularia*, and *Cladosporium*, comprised almost half of the *Ascomycota* (10.0–62.5%; average, 44%). *Eurotiomycetes*, which includes *Aspergillus* and *Penicillium*, accounted for 28% and 24% of the *Ascomycota* sequences in the indoor and outdoor air, respectively. In contrast, the majority of detected *Basidiomycota* species (>95%) belonged to a single class, *Agaricomycetes*. These fungi accounted for 69.5% of the indoor samples and 56.9% of the outdoor samples.

*Hyphodontia* and *Thanatephorus* were the most common fungal genera in the indoor and outdoor air samples (S3 Table). Human skin-associated fungi, such as species belonging to the genus *Malassezia*, were detected in indoor air samples but were nearly absent from the outdoor air samples. Many different types of fungi, including mushrooms (e.g., *Agaricomycetes*), plant pathogens, wood-rotting fungi, and molds, were identified in these air samples.

**Fig 4:**
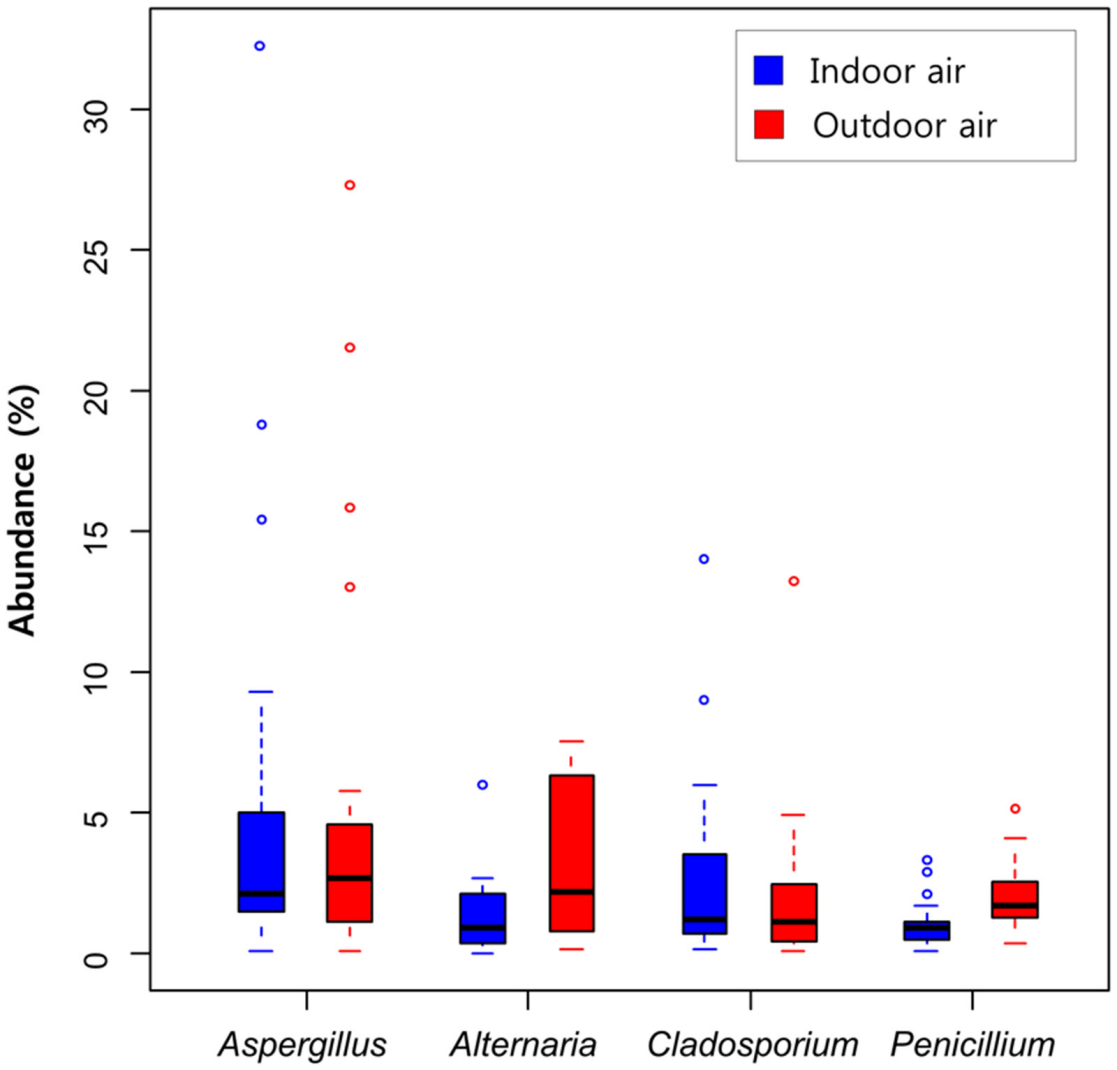
Relative abundance of four allergenic fungi in indoor and outdoor air samples. The solid black lines represent the medians, and the circles are outliers. Bars denote the minimum and maximum values excluding the outliers.

The relative abundance of four representative allergic fungi, namely *Aspergillus*, *Alternaria*, *Cladosporium*, and *Penicillium*, was calculated (Fig 4). The abundance of *Aspergillus* (5.0% indoor; 5.2% outdoor) and *Cladosporium* (2.7% indoor; 2.0% outdoor) did not differ between the indoor and outdoor air. However, the abundance of *Alternaria* (1.3% indoor; 3.2% outdoor) showed a difference (p = 0.0033), and was 2.4 times more abundant in outdoor air than in indoor air. *Penicillium* (1.1% indoor; 1.9% outdoor) was also more abundant (1.8 times; p = 0.0036) in outdoor air than in indoor air.

Because fungal ITS identification varies depending on the database used, the ITS reads obtained in this study were also analyzed by BLAST searches against the UNITE database. Based on the UNITE database, 0.1–66.8% of the ITS reads were identified as uncultured fungi. The fungal genera highly represented in EzFungi were similarly observed in the UNITE database, although their abundances differed (S3 Table). Because the two databases did not yield conflicting results, EzFungi-based taxon composition was used for overall analyses.

### Identification of bacterial species in bioaerosols that are shared with the human microbiome

To identify the bacterial species that originated from humans, the bacterial communities obtained in this study were compared with a previously reported human microbiome. The skin, airways, gastrointestinal tract, oral, and urogenital tract microbiome data reported by Costello *et al*. [61] were used as the reference human microbiome. The proportion of the bacterial communities that came from skin and other human sources were significantly higher (p < 0.0005) in indoor aerosols than in outdoor aerosols (S3 Fig). On average, 10.6% of the bacterial sequences in indoor air came from human skin, and 6.6% or 6.5% of the bacterial sequences in indoor aerosols were from human airways or the oral microbiome, respectively. In comparison, the proportion of human originated bacteria in outdoor samples was lower.

## Discussion

Previous studies of the aerosol microbiology in facilities for children primarily utilized culture-dependent techniques [15,16,19–21,23,62–65]. Despite the development of molecular techniques and the great interest in airborne microbiomes, metagenomic investigations have not been employed to identify the airborne microbes in children’s facilities. Previously, Lee *et al*. [66] investigated the bacterial diversity in a daycare center using culture-independent methods (16S rRNA gene sequencing of clone libraries). They used surface swabs of toys and furniture and found 190 bacterial species. According to the data, the genus *Pseudomonas* was particularly abundant in the clone libraries, followed by uncultured bacterial strains and human-associated bacteria. In the present study, a high abundance of *Pseudomonas* spp. was not observed, which may due to the difference in the sample types. Our results, which were obtained by high throughput sequencing, show that the aerosols in the daytime spaces of children harbor a diverse and dynamic microbial population. Several bacterial genera belonging to the *Proteobacteria* (e.g., *Paracoccus*, *Methylobacterium*, and *Acinetobacter*) were newly identified, as these genera had not been previously reported in schools or daycare centers using cultivation-dependent methods [6]. The high abundance of *Firmicutes* (*Staphylococcus* and *Bacillus*) and *Actinobacteria* (*Corynebacterium* and *Micrococcus*) that was consistently observed in previous culture-based studies was confirmed here.

Traditionally, fungi have been identified by directly observing the morphology of captured spores or after cultivation. Most culture-based fungal studies have focused on detecting several fungal genera (*Alternaria*, *Aspergillus*, *Penicillium*, and *Cladosporium*) and the reported proportion of these genera within air samples varied depending on the study and method of analysis [15,18,23,62,64]. Fungi belonging to the *Ascomycota* can be more easily cultivated than *Basidiomycota* species using traditional media [32,67] and have received attention because of their allergenic effect and clinical importance. In our study, we used the ITS2 region for a metagenomic analysis of fungal communities and identified 855 genera from 50 air samples. The fungal communities were more diverse than the visible communities and contained a wide variety of previously unidentified taxa. Moreover, the dominant species were significantly different from those reported in previous studies using culture-based methods. The dominant fungi from the culture-based studies (*Alternaria*, *Aspergillus*, *Penicillium*, and *Cladosporium*) represented <12% of the total sequence reads, whereas *Basidiomycota* (class *Agaricomycetes*) represented over 60% of the total reads. Similar to the present findings, using ITS1 analysis, Adams *et al*. observed a diverse composition of fungal taxa and a particular abundance of *Agaricomycetes* and *Dothideomycetes* in indoor environments [68–70]. However, another study of the global indoor fungal composition using sequence analysis of the ITS2 region showed a dominant abundance of the phylum *Ascomycota* (particularly class *Dothideomycetes*) [71].

The bacteria in indoor air appeared to be a diverse combination of species associated with both humans and the outside environment. For example, in the indoor air samples, *Micrococcus* and *Staphylococcus* species [72–74], which are commonly found in the human skin microbiome, were observed together with *Paracoccus* [75–77] and *Methylobacterium* species [78,79], which have been isolated from various geographical locations and environments. Additionally, PCoA using weighted UniFrac distances detected a significant difference in the bacterial composition between the two G-In samples and other indoor samples. The G-In samples contained a large proportion of bacterial genera generally detected in outdoor environments. We postulated that the inflow of adjacent outdoor air by the naturally supplied ventilation could have a large impact on indoor air microbiomes as was shown in previous studies [38,80]. The different outdoor environments, various terrains, and land use types (mountain, stream, big market, park, etc.) may explain the differences in the outdoor and/or indoor microbial communities. However, human-associated bacteria greatly contributed to the structure and taxa of the indoor bacterial communities. The source tracking analysis results also support human skin as a primary source of bacteria in indoor air. Moreover, comparison of the bacterial communities that originated from human sources in daycare centers and schools showed that the indoor microbiome varied according to the microbial community of the occupants, similar to what was shown in other studies of indoor surfaces [81–83]. These results were in agreement with a previous report showing that the fundamental source of bacteria in indoor air was the direct shedding of microorganisms from humans during occupancy [40]. Although Meadow *et al*. [43] reported a close resemblance between outdoor and indoor airborne bacterial communities, the human-associated bacterial genera observed in this study were more than twice as abundant in indoor air than in outdoor air. Human occupancy in indoor environments is believed to elevate the concentration of indoor airborne bacteria due to the resuspension of settled dust and the shedding of bacteria from human skin [40,44].

Our study demonstrated that the composition of fungi and bacteria in indoor air is vastly different. In contrast to the bacteria, most fungi detected indoors appeared to originate from diverse outdoor sources, independent of human activity. Known human-associated fungi such as *Malassezia* [84–86], which comprises lipophilic yeasts that are not detected by culturing in common media, were detected in the indoor air; however, the contribution of these fungi to the fungal communities was very low (0.08%). As demonstrated in the UniFrac analyses of this study, the indoor fungal composition did was not differ from the outdoor fungal composition. This result is congruent with the previous report of Adams *et al*. [69], who suggested that residential surfaces are passive collectors of airborne fungi of putative outdoor origin and that aerosolization of endogenous fungi from growth on typical household surfaces is minor compared to the fungal input from outdoors. Other studies using culture-independent techniques also showed that the composition of the indoor fungal community is predominantly of outdoor origin and is geographically patterned [70,71]. It would be not surprising that the human-associated fungal assemblages in indoor air are smaller than the bacterial ones because the total number of fungal cells on humans is orders of magnitude smaller than that of the bacterial microbiota [87].

## Conclusions

Our 454 pyrosequencing approach provided deeper insight into the high diversity of the microbial community in the indoor and outdoor air of spaces typically occupied by children. This study also detected rare airborne microbes not found by traditional culture-based surveys and demonstrated that the airborne microbial communities were likely influenced by various environmental sources. The composition of the indoor bacterial community was influenced by human occupancy; however, the composition of the indoor fungal community originated from diverse outdoor sources rather than from humans. These findings provide a better understanding of the airborne microbes present in human environments and a clue for assessing the infections, asthma, allergy, or other respiratory diseases resulting from exposure to airborne microbes. However, additional analyses at different locations over longer time periods are needed to obtain a comprehensive understanding of the airborne microbiome and the various factors that affect the airborne microbial ecology.

## Supporting Information

S1 Fig Principal coordinate analysis (PCoA) of the fungal community based on (A) the EzFungi database and (B) UNITE database. Differences in the composition of the microbial communities were quantified using a Bray-Curtis dissimilarity matrix.
(TIF)

S2 Fig Relative abundance of four dominant bacterial genera in indoor and outdoor air samples. The solid black lines represent the median, and the circles are outliers. Bars denote the minimum and maximum values excluding the outliers.
(TIF)

S3 Fig The proportion of human originated bacteria in the aerosols. The air sample data were compared to reference human microbiome data, and the proportion of sequences observed in both the sample and reference data was calculated. The most frequently observed human bacteria and their proportion in the indoor aerosols are shown in the lower panel.
(TIF)

S1 Table Sequencing statistics and diversity estimates. (DOCX)

S2 Table Bacterial genera observed in indoor and outdoor air samples. (DOC)

S3 Table Fungal genera observed in indoor and outdoor air samples. (DOCX)

## References

[1] Burrows SM, Elbert W, Lawrence MG, Poschl U. Bacteria in the global atmosphere—Part 1: Review and synthesis of literature data for different ecosystems. Atmos Chem Phys. 2009;9: 9263–9280.

[2] Bauer H, Kasper-Giebl A, Loflund M, Giebl H, Hitzenberger R, Zibuschka F, et al The contribution of bacteria and fungal spores to the organic carbon content of cloud water, precipitation and aerosols. Atmos Res. 2002;64: 109–119. 12106611

[3] Elbert W, Taylor PE, Andreae MO, Poschl U. Contribution of fungi to primary biogenic aerosols in the atmosphere: wet and dry discharged spores, carbohydrates, and inorganic ions. Atm Chem Phys. 2007;7: 4569–4588.

[4] Frohlich-Nowoisky J, Burrows SM, Xie Z, Engling G, Solomon PA, Fraser MP, et al Biogeography in the air: fungal diversity over land and oceans. Biogeosciences. 2012;9: 1125–1136.

[5] Despres VR, Huffman JA, Burrows SM, Hoose C, Safatov AS, Buryak G, et al Primary biological aerosol particles in the atmosphere: a review. Tellus B Chem Phys Meteorol. 2012;64: 1–58.

[6] Gandolfi I, Bertolini V, Ambrosini R, Bestetti G, Franzetti A. Unravelling the bacterial diversity in the atmosphere. Appl Microbiol Biotechnol. 2013;97: 4727–4736. 10.1007/s00253-013-4901-2 23604562

[7] Womack AM, Bohannan BJ, Green JL. Biodiversity and biogeography of the atmosphere. Philos Trans R Soc Lond B Biol Sci. 2010;365: 3645–3653. 10.1098/rstb.2010.0283 20980313 PMC2982008

[8] Zukiewicz-Sobczak WA. The role of fungi in allergic diseases. Postepy Dermatol Alergol. 2013;30: 42–45. 10.5114/pdia.2013.33377 24278044 PMC3834689

[9] Denning DW, O'Driscoll BR, Hogaboam CM, Bowyer P, Niven RM. The link between fungi and severe asthma: a summary of the evidence. Eur Respir J. 2006;27: 615–626. 16507864 10.1183/09031936.06.00074705

[10] Faergemann J. Atopic dermatitis and fungi. Clin Microbiol Rev. 2002;15: 545–563. 12364369 10.1128/CMR.15.4.545-563.2002 PMC126862

[11] Knutsen AP, Bush RK, Demain JG, Denning DW, Dixit A, Fairs A, et al Fungi and allergic lower respiratory tract diseases. J Allergy Clin Immunol. 2012;129: 280–291. 10.1016/j.jaci.2011.12.970 22284927

[12] Crameri R, Garbani M, Rhyner C, Huitema C. Fungi: the neglected allergenic sources. Allergy. 2014;69: 176–185. 10.1111/all.12325 24286281

[13] Suk WA, Murray K, Avakian MD. Environmental hazards to children's health in the modern world. Mutat Res. 2003;544: 235–242. 14644325 10.1016/j.mrrev.2003.06.007

[14] Neri M, Bonassi S, Knudsen LE, Sram RJ, Holland N, Ugolini D, et al Children's exposure to environmental pollutants and biomarkers of genetic damage. I. Overview and critical issues. Mutat Res. 2006;612: 1–13. 16002329 10.1016/j.mrrev.2005.04.001

[15] Aydogdu H, Asan A, Otkun MT, Ture M. Monitoring of fungi and bacteria in the indoor air of primary schools in Edirne city, Turkey. Indoor Built Environ. 2005;14: 411–425.

[16] Aydogdu H, Asan A, Otkun MT. Indoor and outdoor airborne bacteria in child day-care centers in Edirne City (Turkey), seasonal distribution and influence of meteorological factors. Environ Monit Assess. 2010;164: 53–66. 10.1007/s10661-009-0874-0 19404760

[17] Fang Z, Ouyang Z, Zheng H, Wang X, Hu L. Culturable airborne bacteria in outdoor environments in Beijing, China. Microb Ecol. 2007;54: 487–496. 17308950 10.1007/s00248-007-9216-3

[18] Kim KY, Kim YS, Kim D, Kim HT. Exposure level and distribution characteristics of airborne bacteria and fungi in Seoul metropolitan subway stations. Ind Health. 2011;49: 242–248. 21173524 10.2486/indhealth.ms1199

[19] Andersson AM, Weiss N, Rainey F, Salkinoja-Salonen MS. Dust-borne bacteria in animal sheds, schools and children's day care centres. J Appl Microbiol. 1999;86: 622–634. 10212408 10.1046/j.1365-2672.1999.00706.x

[20] Kim KY, Kim CN. Airborne microbiological characteristics in public buildings of Korea. Build Environ. 2007;42: 2188–2196.

[21] Bartlett KH, Kennedy SM, Brauer M, van Netten C, Dill B. Evaluation and determinants of airborne bacterial concentrations in school classrooms. J Occup Environ Hyg. 2004;1: 639–647. 15631055 10.1080/15459620490497744

[22] Shelton BG, Kirkland KH, Flanders WD, Morris GK. Profiles of airborne fungi in buildings and outdoor environments in the United States. Appl Environ Microbiol. 2002;68: 1743–1753. 11916692 10.1128/AEM.68.4.1743-1753.2002 PMC123871

[23] Jo WK, Seo YJ. Indoor and outdoor bioaerosol levels at recreation facilities, elementary schools, and homes. Chemosphere. 2005;61: 1570–1579. 15982704 10.1016/j.chemosphere.2005.04.103

[24] Amann RI, Ludwig W, Schleifer KH. Phylogenetic identification and in situ detection of individual microbial cells without cultivation. Microbiol Rev. 1995;59: 143–169. 7535888 10.1128/mr.59.1.143-169.1995 PMC239358

[25] Hugenholtz P, Goebel BM, Pace NR. Impact of culture-independent studies on the emerging phylogenetic view of bacterial diversity. J Bacteriol. 1998;180: 4765–4774. 9733676 10.1128/jb.180.18.4765-4774.1998 PMC107498

[26] Cho BC, Hwang CY. Prokaryotic abundance and 16S rRNA gene sequences detected in marine aerosols on the East Sea (Korea). FEMS Microbiol Ecol. 2011;76: 327–341. 10.1111/j.1574-6941.2011.01053.x 21255051

[27] Fierer N, Liu Z, Rodriguez-Hernandez M, Knight R, Henn M, Hernandez MT. Short-term temporal variability in airborne bacterial and fungal populations. Appl Environ Microbiol. 2008;74: 200–207. 17981945 10.1128/AEM.01467-07 PMC2223228

[28] Schoch CL, Seifert KA, Huhndorf S, Robert V, Spouge JL, Levesque CA, et al Nuclear ribosomal internal transcribed spacer (ITS) region as a universal DNA barcode marker for Fungi. Proc Natl Acad Sci U S A. 2012;109: 6241–6246. 10.1073/pnas.1117018109 22454494 PMC3341068

[29] Ihrmark K, Bodeker ITM, Cruz-Martinez K, Friberg H, Kubartova AA, Schenck J, et al New primers to amplify the fungal ITS2 region—evaluation by 454-sequencing of artificial and natural communities. FEMS Microbiol Ecol. 2012;82: 666–677. 10.1111/j.1574-6941.2012.01437.x 22738186

[30] Toju H, Tanabe AS, Yamamoto S, Sato H. High-Coverage ITS Primers for the DNA-based identification of Ascomycetes and Basidiomycetes in environmental samples. PLoS One. 2012;7: e40863 10.1371/journal.pone.0040863 22808280 PMC3395698

[31] Yamamoto N, Bibby K, Qian J, Hospodsky D, Rismani-Yazdi H, Nazaroff WW, et al Particle-size distributions and seasonal diversity of allergenic and pathogenic fungi in outdoor air. ISME J. 2012;6: 1801–1811. 10.1038/ismej.2012.30 22476354 PMC3446800

[32] Frohlich-Nowoisky J, Pickersgill DA, Despres VR, Poschl U. High diversity of fungi in air particulate matter. Proc Natl Acad Sci U S A. 2009;106: 12814–12819. 10.1073/pnas.0811003106 19617562 PMC2722276

[33] Tringe SG, Zhang T, Liu X, Yu Y, Lee WH, Yap J, et al The airborne metagenome in an indoor urban environment. PLoS One. 2008;3: e1862 10.1371/journal.pone.0001862 18382653 PMC2270337

[34] Bertolini V, Gandolfi I, Ambrosini R, Bestetti G, Innocente E, Rampazzo G, et al Temporal variability and effect of environmental variables on airborne bacterial communities in an urban area of Northern Italy. Appl Microbiol Biotechnol. 2013;97: 6561–6570. 10.1007/s00253-012-4450-0 23053100

[35] Bowers RM, Sullivan AP, Costello EK, Collett JL Jr., Knight R, Fierer N. Sources of bacteria in outdoor air across cities in the midwestern United States. Appl Environ Microbiol. 2011;77: 6350–6356. 10.1128/AEM.05498-11 21803902 PMC3187178

[36] Brodie EL, DeSantis TZ, Parker JP, Zubietta IX, Piceno YM, Andersen GL. Urban aerosols harbor diverse and dynamic bacterial populations. Proc Natl Acad Sci U S A. 2007;104: 299–304. 17182744 10.1073/pnas.0608255104 PMC1713168

[37] Robertson CE, Baumgartner LK, Harris JK, Peterson KL, Stevens MJ, Frank DN, et al Culture-independent analysis of aerosol microbiology in a metropolitan subway system. Appl Environ Microbiol. 2013;79: 3485–3493. 10.1128/AEM.00331-13 23542619 PMC3648054

[38] Leung MHY, Wilkins D, Li EKT, Kong FKF, Lee PKH. Indoor-air microbiome in an urban subway network: diversity and dynamics. Appl Environ Microbiol. 2014;80: 6760–6770. 10.1128/AEM.02244-14 25172855 PMC4249038

[39] Gauzere C, Moletta-Denat M, Blanquart H, Ferreira S, Moularat S, Godon JJ, et al Stability of airborne microbes in the Louvre Museum over time. Indoor Air. 2014;24: 29–40. 10.1111/ina.12053 23710880

[40] Hospodsky D, Qian J, Nazaroff WW, Yamamoto N, Bibby K, Rismani-Yazdi H, et al Human occupancy as a source of indoor airborne bacteria. PLoS One. 2012;7(4):e34867 10.1371/journal.pone.0034867 22529946 PMC3329548

[41] Dziewit L, Czarnecki J, Wibberg D, Radlinska M, Mrozek P, Szymczak M, et al Architecture and functions of a multipartite genome of the methylotrophic bacterium *Paracoccus aminophilus* JCM 7686, containing primary and secondary chromids. BMC Genomics. 2014;15: 124 10.1186/1471-2164-15-124 24517536 PMC3925955

[42] Yooseph S, Andrews-Pfannkoch C, Tenney A, McQuaid J, Williamson S, Thiagarajan M, et al A metagenomic framework for the study of airborne microbial communities. PLoS One. 2013;8: e81862 10.1371/journal.pone.0081862 24349140 PMC3859506

[43] Meadow JF, Altrichter AE, Kembel SW, Kline J, Mhuireach G, Moriyama M, et al Indoor airborne bacterial communities are influenced by ventilation, occupancy, and outdoor air source. Indoor Air. 2014;24: 41–48. 10.1111/ina.12047 23621155 PMC4285785

[44] Qian J, Hospodsky D, Yamamoto N, Nazaroff WW, Peccia J. Size-resolved emission rates of airborne bacteria and fungi in an occupied classroom. Indoor Air. 2012;22: 339–351. 10.1111/j.1600-0668.2012.00769.x 22257156 PMC3437488

[45] Chun J, Kim KY, Lee JH, Choi Y. The analysis of oral microbial communities of wild-type and toll-like receptor 2-deficient mice using a 454 GS FLX Titanium pyrosequencer. BMC Microbiol. 2010;10: 101 10.1186/1471-2180-10-101 20370919 PMC2873484

[46] Schoch CL, Seifert KA, Huhndorf S, Robert V, Spouge JL, Levesque CA, et al Nuclear ribosomal internal transcribed spacer (ITS) region as a universal DNA barcode marker for Fungi. Proc Natl Acad Sci U S A. 2012;109: 6241–6246. 10.1073/pnas.1117018109 22454494 PMC3341068

[47] Hur M, Kim Y, Song HR, Kim JM, Choi YI, Yi H. Effect of genetically modified poplars on soil microbial communities during the phytoremediation of waste mine tailings. Appl Environ Microbiol. 2011;77: 7611–7619. 10.1128/AEM.06102-11 21890678 PMC3209168

[48] Yi H, Yong D, Lee K, Cho YJ, Chun J. Profiling bacterial community in upper respiratory tracts. BMC Infect Dis. 2014;14: 583 10.1186/s12879-014-0583-3 25391813 PMC4236460

[49] Jeon YS, Chun J, Kim BS. Identification of household bacterial community and analysis of species shared with human microbiome. Curr Microbiol. 2013;67: 557–563. 10.1007/s00284-013-0401-y 23743600 PMC3790245

[50] Eddy SR. Accelerated profile HMM searches. PLoS Comput Biol. 2011;7: e1002195 10.1371/journal.pcbi.1002195 22039361 PMC3197634

[51] Lee JH, Yi H, Jeon YS, Won S, Chun J. TBC: a clustering algorithm based on prokaryotic taxonomy. J Microbiol. 2012;50: 181–185. 10.1007/s12275-012-1214-6 22538644

[52] Kim OS, Cho YJ, Lee K, Yoon SH, Kim M, Na H, et al Introducing EzTaxon-e: a prokaryotic 16S rRNA gene sequence database with phylotypes that represent uncultured species. Int J Syst Evol Microbiol. 2012;62: 716–721. 10.1099/ijs.0.038075-0 22140171

[53] Myers EW, Miller W. Optimal alignments in linear space. Comput Appl Biosci. 1988;4: 11–17. 3382986 10.1093/bioinformatics/4.1.11

[54] Higgins DG, Sharp PM Fast and sensitive multiple sequence alignments on a microcomputer. Comput Appl Biosci. 1989;5: 151–153. 2720464 10.1093/bioinformatics/5.2.151

[55] Edgar RC, Haas BJ, Clemente JC, Quince C, Knight R. UCHIME improves sensitivity and speed of chimera detection. Bioinformatics. 2011;27: 2194–2200. 10.1093/bioinformatics/btr381 21700674 PMC3150044

[56] Fahlgren C, Hagstrom A, Nilsson D, Zweifel UL. Annual variations in the diversity, viability, and origin of airborne bacteria. Appl Environ Microbiol. 2010;76: 3015–3025. 10.1128/AEM.02092-09 20228096 PMC2863461

[57] Chao A, Bunge J. Estimating the number of species in a stochastic abundance model. Biometrics. 2002;58: 531–539. 12229987 10.1111/j.0006-341x.2002.00531.x

[58] Shannon CE, Weaver W. The mathematical theory of communication. Urbana, IL: University of Illinois Press; 1949.

[59] Hill TCJ, Walsh KA, Harris JA, Moffett BF. Using ecological diversity measures with bacterial communities. FEMS Microbiol Ecol. 2003;43: 1–11. 10.1111/j.1574-6941.2003.tb01040.x 19719691

[60] Storey JD. A direct approach to false discovery rates. J R Stat Soc Series B Stat Methodol. 2002;64: 479–498.

[61] Costello EK, Lauber CL, Hamady M, Fierer N, Gordon JI, Knight R. Bacterial community variation in human body habitats across space and time. Science. 2009;326: 1694–1697. 10.1126/science.1177486 19892944 PMC3602444

[62] Kim JL, Elfman L, Mi Y, Wieslander G, Smedje G, Norbäck D. Indoor molds, bacteria, microbial volatile organic compounds and plasticizers in schools—associations with asthma and respiratory symptoms in pupils. Indoor Air. 2007;17: 153–163. 17391238 10.1111/j.1600-0668.2006.00466.x

[63] Lis DO, Gorny RL. Haemophilus influenzae as an airborne contamination in child day care centers. Am J Infect Control. 2013;41: 438–442. 10.1016/j.ajic.2012.05.023 22980511

[64] Roda C, Barral S, Ravelomanantsoa H, Dusseaux M, Tribout M, Le Moullec Y, et al Assessment of indoor environment in Paris child day care centers. Environ Res. 2011;111: 1010–1017. 10.1016/j.envres.2011.06.009 21783190

[65] Chen NT, Su YM, Hsu NY, Wu PC, Su HJ. Airborne fungi and bacteria in child daycare centers and the effectiveness of weak acid hypochlorous water on controlling microbes. J Environ Monit. 2012;14: 2692–2697. 22907131 10.1039/c2em30113j

[66] Lee L, Tin S, Kelley ST. Culture-independent analysis of bacterial diversity in a child-care facility. BMC Microbiol. 2007;7: 27 17411442 10.1186/1471-2180-7-27 PMC1853100

[67] Bridge P, Spooner B. Soil fungi: diversity and detection. Plant Soil. 2001;232: 147–154.

[68] Adams RI, Amend AS, Taylor JW, Bruns TD. A unique signal distorts the perception of species richness and composition in high-throughput sequencing surveys of microbial communities: a case study of fungi in indoor dust. Microb Ecol. 2013;66: 735–741. 10.1007/s00248-013-0266-4 23880792 PMC3824195

[69] Adams RI, Miletto M, Taylor JW, Bruns TD. The diversity and distribution of fungi on residential surfaces. PLoS One. 2013;8: e78866 10.1371/journal.pone.0078866 24223861 PMC3815347

[70] Adams RI, Miletto M, Taylor JW, Bruns TD. Dispersal in microbes: fungi in indoor air are dominated by outdoor air and show dispersal limitation at short distances. ISME J. 2013;7: 1262–1273. 10.1038/ismej.2013.28 23426013 PMC3695294

[71] Amend AS, Seifert KA, Samson R, Bruns TD. Indoor fungal composition is geographically patterned and more diverse in temperate zones than in the tropics. Proc Natl Acad Sci U S A. 2010;107: 13748–13753. 10.1073/pnas.1000454107 20616017 PMC2922287

[72] Kloos WE, Musselwhite MS. Distribution and persistence of *Staphylococcus* and *Micrococcus* species and other aerobic bacteria on human skin. Appl Microbiol. 1975;30: 381–395. 810086 10.1128/am.30.3.381-395.1975 PMC187193

[73] Noble WC. Skin microbiology: coming of age. J Med Microbiol. 1984;17: 1–12. 6229637 10.1099/00222615-17-1-1

[74] Human Microbiome Project C. A framework for human microbiome research. Nature. 2012;486: 215–221. 10.1038/nature11209 22699610 PMC3377744

[75] Kampfer P, Lai WA, Arun AB, Young CC, Rekha PD, Martin K, et al *Paracoccus rhizosphaerae* sp. nov., isolated from the rhizosphere of the plant *Crossostephium chinense* (L.) Makino (Seremban). Int J Syst Evol Microbiol. 2012;62: 2750–2756. 10.1099/ijs.0.039057-0 22286908

[76] Khan ST, Takaich S, Harayamal S. *Paracoccus marinus* sp. nov., an adonixanthin diglucoside-producing bacterium isolated from coastal seawater in Tokyo Bay. Int J Syst Evol Microbiol. 2008;58: 383–386. 10.1099/ijs.0.65103-0 18218935

[77] Urakami T, Araki H, Oyanagi H, Suzuki KI, Komagata K. *Paracoccus Aminophilus* sp. nov. and *Paracoccus Aminovorans* sp. nov., which utilize N,N-Dimethylformamide. Int J Syst Bacteriol. 1990;40: 287–291. 2397196 10.1099/00207713-40-3-287

[78] Veyisoglu A, Camas M, Tatar D, Guven K, Sazaki A, Sahin N. *Methylobacterium tarhaniae* sp. nov., isolated from arid soil. Int J Syst Evol Microbiol. 2013;63: 2823–2828. 10.1099/ijs.0.049551-0 23315404

[79] Wellner S, Lodders N, Glaeser SP, Kampfer P. *Methylobacterium trifolii* sp. nov. and *Methylobacterium thuringiense* sp. nov., methanol-utilizing, pink-pigmented bacteria isolated from leaf surfaces. Int J Syst Evol Microbiol. 2013;63: 2690–2699. 10.1099/ijs.0.047787-0 23291886

[80] Kembel SW, Meadow JF, O'Connor TK, Mhuireach G, Northcutt D, Kline J, et al Architectural design drives the biogeography of indoor bacterial communities. PLoS One. 2014;29;9(1):e87093 10.1371/journal.pone.0087093 24489843 PMC3906134

[81] Lax S, Smith DP, Hampton-Marcell J, Owens SM, Handley KM, Scott NM, et al Longitudinal analysis of microbial interaction between humans and the indoor environment. Science. 2014;345: 1048–1052. 10.1126/science.1254529 25170151 PMC4337996

[82] Hewitt KM, Mannino FL, Gonzalez A, Chase JH, Caporaso JG, Knight R, et al Bacterial diversity in two neonatal intensive care units (NICUs). PLoS One. 2013;8(1):e54703 10.1371/journal.pone.0054703 23372757 PMC3553055

[83] Flores GE, Bates ST, Knights D, Lauber CL, Stombaugh J, Knight R, et al Microbial biogeography of public restroom surfaces. PLoS One. 2011;6(11):e28132 10.1371/journal.pone.0028132 22132229 PMC3223236

[84] Gupta AK, Batra R, Bluhm R, Boekhout T, Dawson TL Jr., Skin diseases associated with *Malassezia* species. J Am Acad Dermatol. 2004;51: 785–798. 15523360 10.1016/j.jaad.2003.12.034

[85] Saunders CW, Scheynius A, Heitman J. *Malassezia* fungi are specialized to live on skin and associated with dandruff, eczema, and other skin diseases. PLoS Pathog. 2012;8: e1002701 10.1371/journal.ppat.1002701 22737067 PMC3380954

[86] Gaitanis G, Magiatis P, Hantschke M, Bassukas ID, Velegraki A. The *Malassezia* genus in skin and systemic diseases. Clin Microbiol Rev. 2012;25: 106–141. 10.1128/CMR.00021-11 22232373 PMC3255962

[87] Huffnagle GB, Noverr MC. The emerging world of the fungal microbiome. Trends Microbiol. 2013;21: 334–341. 10.1016/j.tim.2013.04.002 23685069 PMC3708484

