# Supplementary figures and images for: Metagenomic Insights into the Bioaerosols in the Indoor and Outdoor Environments of Childcare Facilities
Source: PLoS One. 2015 May 28;10(5):e0126960. doi: 10.1371/journal.pone.0126960 (PMC4447338; doi:10.1371/journal.pone.0126960)

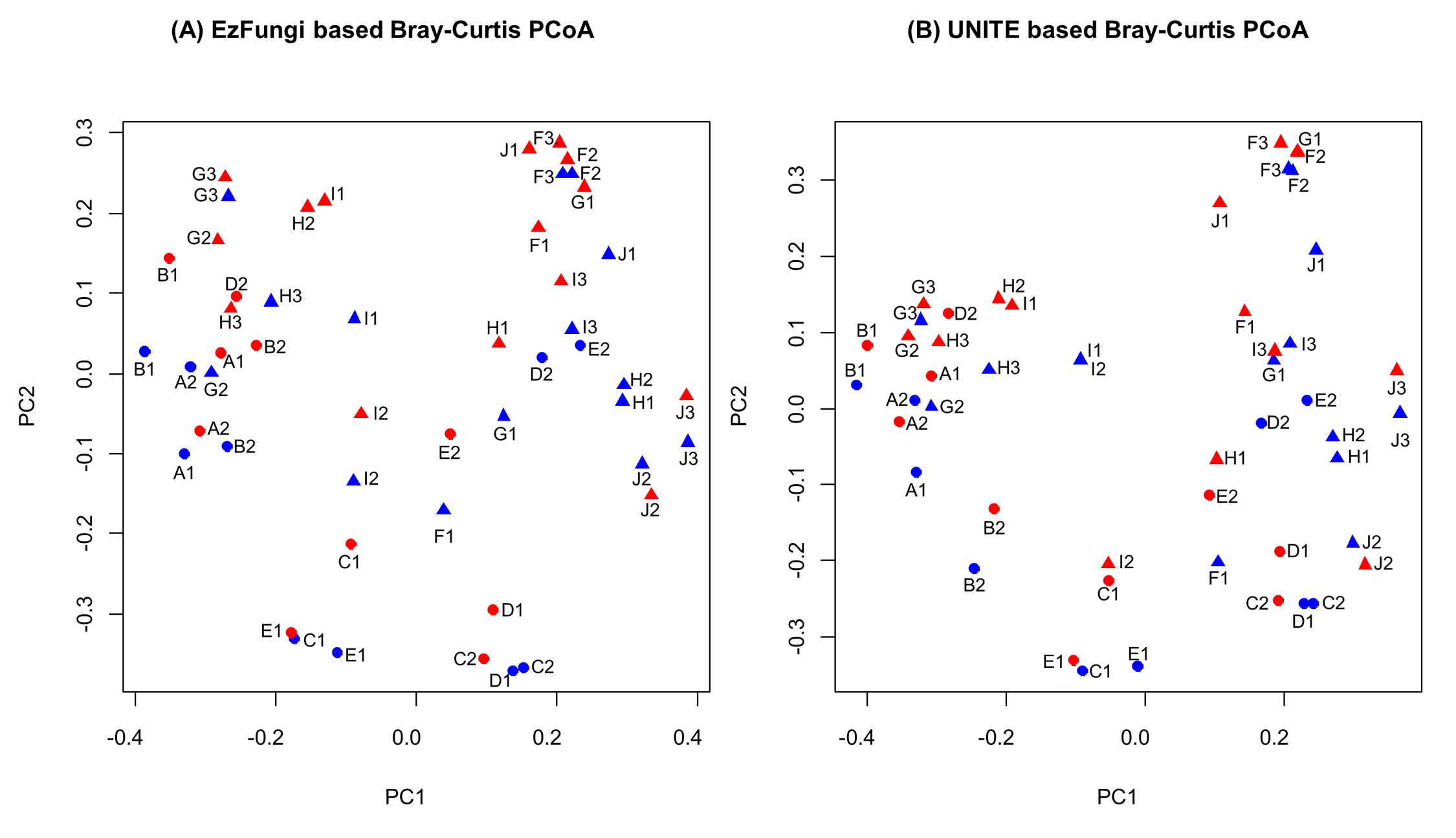

Supplement: S1 Fig — Differences in the composition of the microbial communities were quantified using a Bray-Curtis dissimilarity matrix. (TIF) [file pone.0126960.s001.tif]

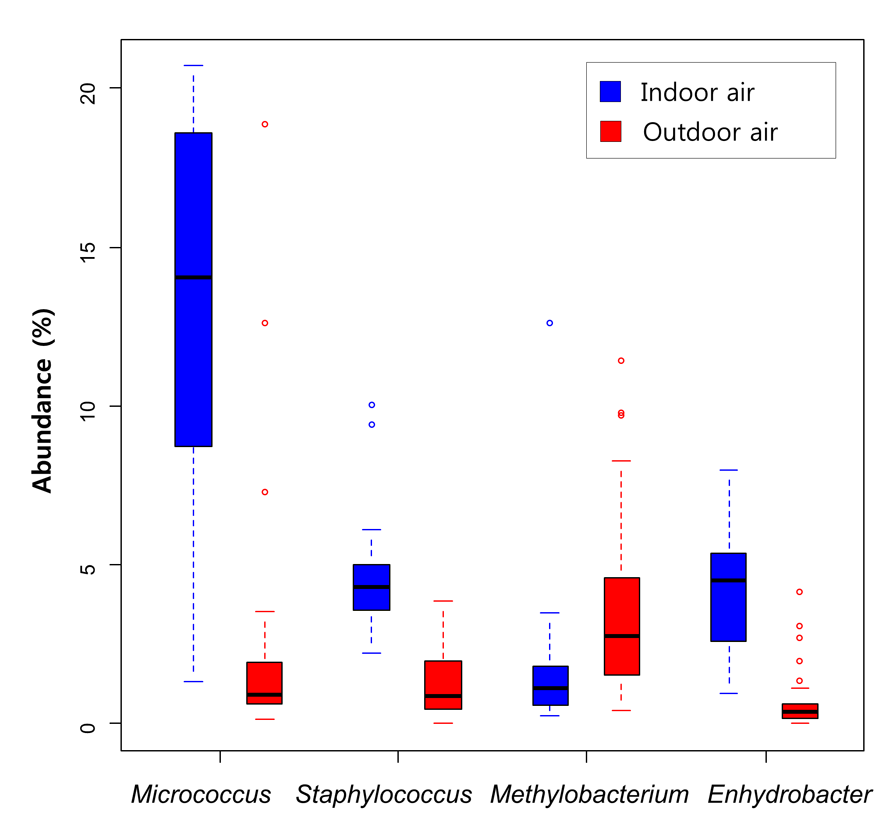

Supplement: S2 Fig — The solid black lines represent the median, and the circles are outliers. Bars denote the minimum and maximum values excluding the outliers. (TIF) [file pone.0126960.s002.tif]

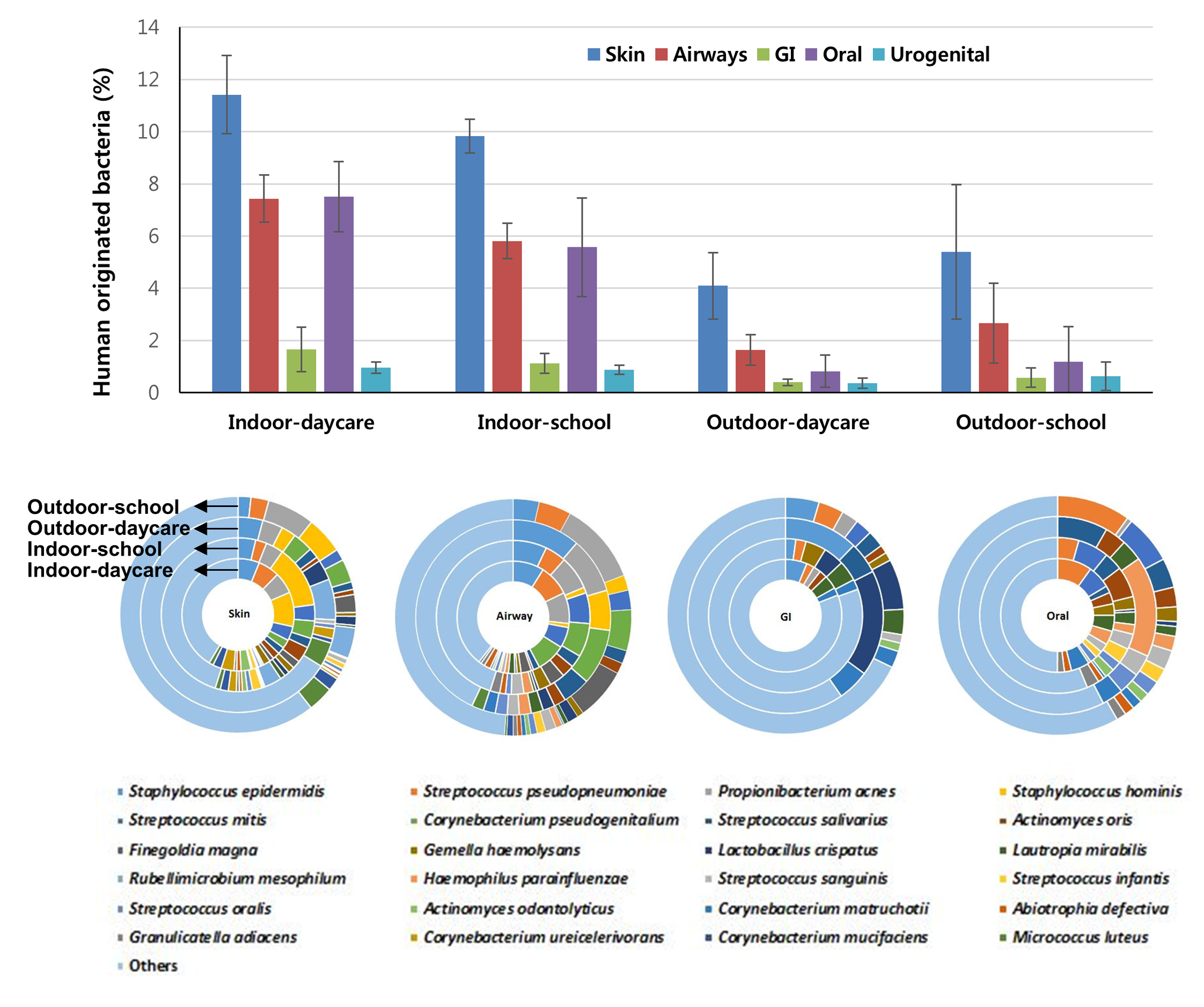

Supplement: S3 Fig — The air sample data were compared to reference human microbiome data, and the proportion of sequences observed in both the sample and reference data was calculated. The most frequently observed human bacteria and their proportion in the indoor aerosols are shown in the lower panel. (TIF) [file pone.0126960.s003.tif]
