# Supplementary material for: Metagenomic Insights into the Bioaerosols in the Indoor and Outdoor Environments of Childcare Facilities
Source: PLoS One. 2015 May 28;10(5):e0126960. doi: 10.1371/journal.pone.0126960 (PMC4447338; doi:10.1371/journal.pone.0126960)
Supplement: S1 Table — (DOCX) [file pone.0126960.s004.docx]

**S1 Table. Sequencing statistics and diversity estimates**

| Sample ID |  | Bacteria | | | | |  |  | | Fungi | | | |
| --- | --- | --- | --- | --- | --- | --- | --- | --- | --- | --- | --- | --- | --- |
|  | Valid reads | | Normalized reads | No. of  OTUs | Chao1 estimated | Shannon index |  | Valid reads | Normalized reads | | No. of  OTUs | Chao1 estimated | Shannon index |
| A1-In | 7,359 | | 3,000 | 579 | 1,178 | 4.65 |  | 7,339 | 2,000 | | 512 | 1,076 | 5.09 |
| A2-In | 7,268 | | 3,000 | 530 | 1,041 | 4.58 |  | 5,321 | 2,000 | | 504 | 1,159 | 5.06 |
| B1-In | 4,489 | | 3,000 | 649 | 1,223 | 5.01 |  | 5,242 | 2,000 | | 475 | 973 | 4.96 |
| B2-In | 4,189 | | 3,000 | 750 | 1,291 | 5.30 |  | 5,049 | 2,000 | | 417 | 960 | 4.54 |
| C1-In | 8,185 | | 3,000 | 638 | 1,634 | 4.74 |  | 4,876 | 2,000 | | 460 | 1,170 | 4.70 |
| C2-In | 1,680 | | 1,559 | 449 | 697 | 5.24 |  | 8,256 | 2,000 | | 381 | 875 | 3.89 |
| D1-In | 531 | | 528 | 189 | 348 | 4.51 |  | 8,604 | 2,000 | | 313 | 786 | 3.38 |
| D2-In | 4,301 | | 3,000 | 529 | 967 | 4.56 |  | 4,777 | 2,000 | | 690 | 1,739 | 5.60 |
| E1-In | 10,771 | | 3,000 | 744 | 1,687 | 5.21 |  | 2,465 | 2,000 | | 429 | 984 | 4.61 |
| E2-In | 11,390 | | 3,000 | 556 | 1,298 | 4.61 |  | 2,013 | 2,000 | | 574 | 1,223 | 4.98 |
| F1-In | 1,774 | | 1,655 | 401 | 622 | 4.81 |  | 4,227 | 2,000 | | 580 | 1,298 | 5.26 |
| F2-In | 2,872 | | 2,630 | 653 | 1,071 | 5.23 |  | 1,955 | 2,000 | | 417 | 1,054 | 5.27 |
| F3-In | 552 | | 509 | 210 | 334 | 4.75 |  | 4,324 | 2,000 | | 677 | 1,641 | 5.62 |
| G1-In | 1,857 | | 1,765 | 609 | 1,021 | 5.78 |  | 3,006 | 2,000 | | 600 | 1,346 | 5.38 |
| G2-In | 3,180 | | 3,000 | 888 | 1,442 | 6.02 |  | 6,730 | 1,000 | | 463 | 950 | 5.07 |
| G3-In | 1,158 | | 1,168 | 370 | 647 | 5.04 |  | 2,205 | 2,000 | | 565 | 1,030 | 5.55 |
| H1-In | 7,116 | | 3,000 | 998 | 1,924 | 5.75 |  | 3,159 | 2,000 | | 712 | 1,819 | 5.49 |
| H2-In | 7,800 | | 3,000 | 902 | 1,794 | 5.64 |  | 990 | 500 | | 232 | 571 | 4.73 |
| H3-In | 6,538 | | 3,000 | 905 | 1,590 | 5.65 |  | 422 | 200 | | 113 | 271 | 4.41 |
| I1-In | 4,951 | | 3,000 | 1,077 | 2,047 | 6.18 |  | 952 | 500 | | 151 | 326 | 4.51 |
| I2-In | 12,087 | | 3,000 | 884 | 2,167 | 5.42 |  | 780 | 350 | | 271 | 497 | 4.82 |
| I3-In | 1,181 | | 1,126 | 454 | 835 | 5.54 |  | 3,726 | 2,000 | | 595 | 1,273 | 5.07 |
| J1-In | 3,634 | | 3,000 | 956 | 1,617 | 5.80 |  | 5,173 | 2,000 | | 464 | 1,148 | 4.43 |
| J2-In | 4,673 | | 3,000 | 887 | 1,771 | 5.35 |  | 3,517 | 2,000 | | 439 | 1,182 | 4.28 |
| J3-In | 5,750 | | 3,000 | 759 | 1,405 | 5.24 |  | 1,638 | 800 | | 171 | 377 | 3.39 |

**S1 Table. Continued.**

| Sample ID |  | Bacteria | | | | |  |  | | Fungi | | | |
| --- | --- | --- | --- | --- | --- | --- | --- | --- | --- | --- | --- | --- | --- |
|  | Valid reads | | Normalized reads | No. of  OTUs | Chao1 estimated | Shannon index |  | Valid reads | Normalized reads | | No. of  OTUs | Chao1 estimated | Shannon index |
| A1-Out | 6,164 | | 3,000 | 883 | 1,413 | 6.00 |  | 4,299 | 2,000 | | 330 | 860 | 3.64 |
| A2-Out | 3,344 | | 2,943 | 938 | 1,531 | 6.09 |  | 4,818 | 2,000 | | 285 | 800 | 3.13 |
| B1-Out | 2,337 | | 2,238 | 673 | 1,059 | 5.78 |  | 4,389 | 2,000 | | 477 | 963 | 5.13 |
| B2-Out | 3,414 | | 3,000 | 768 | 1,293 | 5.77 |  | 1,501 | 700 | | 225 | 507 | 4.53 |
| C1-Out | 4,314 | | 3,000 | 959 | 1,553 | 6.26 |  | 5,984 | 2,000 | | 480 | 996 | 5.00 |
| C2-Out | 2,449 | | 2,182 | 455 | 730 | 5.31 |  | 4,853 | 2,000 | | 333 | 777 | 3.65 |
| D1-Out | 474 | | 453 | 106 | 172 | 3.90 |  | 2,907 | 2,000 | | 309 | 792 | 3.56 |
| D2-Out | 2,213 | | 1,917 | 264 | 420 | 4.68 |  | 4,617 | 2,000 | | 491 | 1,187 | 5.08 |
| E1-Out | 5,293 | | 3,000 | 943 | 1,546 | 6.02 |  | 7,909 | 2,000 | | 416 | 926 | 4.48 |
| E2-Out | 5,860 | | 2,794 | 820 | 1,338 | 5.93 |  | 2,280 | 2,000 | | 478 | 1,120 | 4.82 |
| F1-Out | 7,478 | | 3,000 | 650 | 1,359 | 4.79 |  | 2,412 | 2,000 | | 459 | 1,364 | 4.57 |
| F2-Out | 23,170 | | 3,000 | 584 | 1,761 | 4.02 |  | 1,268 | 600 | | 253 | 632 | 4.91 |
| F3-Out | 8,507 | | 3,000 | 823 | 1,742 | 5.28 |  | 5,214 | 2,000 | | 612 | 1,323 | 5.46 |
| G1-Out | 3,388 | | 2,506 | 603 | 950 | 5.63 |  | 2,861 | 2,000 | | 483 | 896 | 4.92 |
| G2-Out | 3,908 | | 3,000 | 411 | 679 | 4.67 |  | 5,680 | 2,000 | | 489 | 1,121 | 4.91 |
| G3-Out | 2,651 | | 2,516 | 662 | 1,049 | 5.69 |  | 1,638 | 800 | | 258 | 437 | 4.92 |
| H1-Out | 4,731 | | 3,000 | 1,109 | 1,780 | 6.48 |  | 1,175 | 500 | | 199 | 428 | 4.62 |
| H2-Out | 6,984 | | 3,000 | 1,448 | 3,563 | 6.59 |  | 2,939 | 2,000 | | 450 | 921 | 4.88 |
| H3-Out | 5,910 | | 3,000 | 1,584 | 4,055 | 6.94 |  | 5,699 | 2,000 | | 454 | 925 | 4.51 |
| I1-Out | 4,871 | | 3,000 | 1,097 | 2,166 | 6.36 |  | 2,381 | 2,000 | | 327 | 693 | 3.62 |
| I2-Out | 5,044 | | 3,000 | 1,096 | 1,899 | 6.40 |  | 4,140 | 2,000 | | 462 | 807 | 5.14 |
| I3-Out | 5,584 | | 3,000 | 879 | 1,530 | 6.04 |  | 2,861 | 2,000 | | 485 | 1,009 | 4.40 |
| J1-Out | 3,940 | | 3,000 | 757 | 1,242 | 5.74 |  | 5,395 | 2,000 | | 469 | 1,189 | 4.67 |
| J2-Out | 4,572 | | 3,000 | 839 | 1,705 | 5.11 |  | 6,395 | 2,000 | | 461 | 1,098 | 4.56 |
| J3-Out | 2,885 | | 2,820 | 647 | 924 | 5.49 |  | 4,731 | 2,000 | | 379 | 751 | 4.07 |
