# Supplementary material for: Metagenomic Insights into the Bioaerosols in the Indoor and Outdoor Environments of Childcare Facilities
Source: PLoS One. 2015 May 28;10(5):e0126960. doi: 10.1371/journal.pone.0126960 (PMC4447338; doi:10.1371/journal.pone.0126960)
Supplement: S2 Table — (DOC) [file pone.0126960.s005.doc]

**S2 Table. Bacterial genera observed in indoor and outdoor air samples.**

| Bacterial Genera | Indoor air | |  | Outdoor air | | p-value  (In vs. Out) |
| --- | --- | --- | --- | --- | --- | --- |
|  | Daycare center (n=5x2) | Elementary school (n=5x3) |  | Daycare center (n=5x2) | Elementary school (n=5x3) |  |
| *Micrococcus* | 14.6258 | 12.2007 |  | 1.4673 | 3.2113 | 1E-09 |
| *Paracoccus* | 7.9823 | 3.3212 |  | 1.7957 | 1.6387 | 4E-06 |
| *Staphylococcus* | 5.2757 | 4.1868 |  | 1.0570 | 1.3961 | 2E-09 |
| *Methylobacterium* | 0.8811 | 2.2543 |  | 3.8750 | 3.5593 | 2E-02 |
| *Enhydrobacter* | 4.8833 | 3.9123 |  | 0.5227 | 0.9807 | 7E-09 |
| *Corynebacterium* | 3.3914 | 3.1190 |  | 1.8921 | 1.3944 | 2E-05 |
| *Bacillus* | 2.3951 | 0.9531 |  | 4.6137 | 1.7901 | 5E-02 |
| *Streptococcus* | 5.6740 | 2.9050 |  | 0.3511 | 0.6312 | 1E-06 |
| *Acinetobacter* | 2.1782 | 2.7528 |  | 1.7983 | 2.0833 | 4E-01 |
| *Streptomyces* | 0.7052 | 1.8261 |  | 1.9324 | 4.0905 | 4E-02 |
| *Sphingomonas* | 1.2384 | 1.7101 |  | 2.8956 | 2.4274 | 9E-03 |
| *Kocuria* | 3.8137 | 1.3171 |  | 1.7684 | 0.8713 | 1E-01 |
| *Massilia* | 1.6490 | 1.5398 |  | 1.8020 | 1.1166 | 5E-01 |
| *Pseudonocardia* | 0.2417 | 0.6196 |  | 0.5556 | 3.9002 | 8E-02 |
| *Nocardioides* | 0.2756 | 1.5682 |  | 1.4182 | 1.7789 | 8E-02 |
| *Rubellimicrobium* | 0.3560 | 1.1561 |  | 2.1346 | 1.3443 | 8E-03 |
| *Nocardiopsis* | 0.1394 | 0.4602 |  | 0.3530 | 3.5758 | 1E-01 |
| *Blastococcus* | 0.6137 | 0.6823 |  | 1.7460 | 1.4111 | 3E-04 |
| *Skermanella* | 1.4724 | 0.7537 |  | 0.6940 | 1.1451 | 8E-01 |
| *Microbacterium* | 0.5803 | 0.8350 |  | 1.5333 | 1.0715 | 9E-03 |
| *Roseomonas* | 1.6867 | 0.9603 |  | 0.7988 | 0.4175 | 1E-04 |
| *Janibacter* | 1.3251 | 1.3240 |  | 0.4314 | 0.7789 | 2E-03 |
| *Dietzia* | 1.3364 | 1.0817 |  | 0.4546 | 0.7972 | 1E-02 |
| *Clostridium* | 0.3360 | 0.5280 |  | 1.7983 | 0.8721 | 4E-03 |
| *Deinococcus* | 0.5717 | 1.0520 |  | 1.1193 | 0.7251 | 9E-01 |
| *Brevundimonas* | 1.2815 | 1.0413 |  | 0.4549 | 0.4747 | 2E-05 |
| *Pseudomonas* | 0.8478 | 1.0664 |  | 0.6550 | 0.5707 | 1E-01 |
| *Propionibacterium* | 1.2271 | 0.8650 |  | 0.2796 | 0.4225 | 6E-05 |
| *Arthrobacter* | 0.4292 | 0.4969 |  | 0.6684 | 1.0249 | 1E-03 |
| *Brachybacterium* | 0.4894 | 0.4324 |  | 0.4067 | 1.1424 | 1E-01 |
| *Actinomyces* | 0.9921 | 1.0341 |  | 0.0627 | 0.2160 | 2E-08 |
| *Turicibacter* | 0.1752 | 0.1615 |  | 1.1715 | 0.5626 | 2E-03 |
| *Amaricoccus* | 1.0953 | 0.5800 |  | 0.1427 | 0.1936 | 3E-05 |
| *Tumebacillus* | 0.0185 | 0.1971 |  | 0.0907 | 1.7043 | 3E-01 |
| *Gordonia* | 0.6573 | 0.6550 |  | 0.2915 | 0.3668 | 2E-02 |
| *Haemophilus* | 0.7147 | 0.5093 |  | 0.1613 | 0.0639 | 1E-07 |
| *Lactobacillus* | 0.3193 | 0.2687 |  | 0.4415 | 0.2956 | 4E-01 |
| *Neisseria* | 0.6340 | 0.4868 |  | 0.0400 | 0.1001 | 8E-07 |
| *Mycobacterium* | 0.2956 | 0.2764 |  | 0.3035 | 0.2966 | 8E-01 |
| *Enterobacter* | 0.2012 | 0.1874 |  | 0.1720 | 0.2508 | 8E-01 |
| *Escherichia* | 0.0943 | 0.3228 |  | 0.1157 | 0.1503 | 2E-01 |
| *Aerococcus* | 0.2566 | 0.2283 |  | 0.0254 | 0.0711 | 3E-03 |
| *Bacteroides* | 0.2768 | 0.1026 |  | 0.0719 | 0.0430 | 2E-02 |
| *Moraxella* | 0.1846 | 0.1274 |  | 0.0072 | 0.0206 | 4E-04 |
| *Klebsiella* | 0.0927 | 0.1014 |  | 0.0841 | 0.0416 | 4E-01 |
