# Supplementary material for: Metagenomic Insights into the Bioaerosols in the Indoor and Outdoor Environments of Childcare Facilities
Source: PLoS One. 2015 May 28;10(5):e0126960. doi: 10.1371/journal.pone.0126960 (PMC4447338; doi:10.1371/journal.pone.0126960)
Supplement: S3 Table — (DOCX) [file pone.0126960.s006.docx]

**S3 Table. Fungal genera observed in indoor and outdoor air samples.**

| Fungal Genera | Indoor | | | | |  | Outdoor | | | | |
| --- | --- | --- | --- | --- | --- | --- | --- | --- | --- | --- | --- |
|  | Daycare center | |  | Elementary school | |  | Daycare center | |  | Elementary school | |
|  | EzFungi | UNITE |  | EzFungi | UNITE |  | EzFungi | UNITE |  | EzFungi | UNITE |
| *Hyphodontia* | 15.02 | 3.69 |  | 4.26 | 1.35 |  | 21.79 | 3.24 |  | 1.73 | 0.59 |
| *Thanatephorus* | 12.64 | 1.08 |  | 9.46 | 0.88 |  | 9.73 | 0.79 |  | 6.54 | 0.59 |
| *Aspergillus* | 7.20 | 4.42 |  | 3.54 | 2.17 |  | 4.07 | 2.27 |  | 5.98 | 3.96 |
| *Perenniporia* | 4.16 | 0.67 |  | 3.32 | 0.75 |  | 6.17 | 0.88 |  | 4.67 | 0.46 |
| *Irpex* | 6.51 | 3.50 |  | 3.80 | 1.80 |  | 4.86 | 3.32 |  | 1.92 | 1.22 |
| *Trametes* | 4.03 | 3.62 |  | 5.90 | 3.89 |  | 2.14 | 2.13 |  | 3.11 | 2.16 |
| *Phlebia* | 2.98 | 3.42 |  | 5.16 | 4.87 |  | 1.69 | 2.24 |  | 3.63 | 2.99 |
| *Antrodiella* | 1.66 | 1.51 |  | 6.83 | 6.37 |  | 0.73 | 0.67 |  | 4.14 | 3.92 |
| *Eukarya_uc* | 1.69 | 1.80 |  | 2.64 | 3.39 |  | 2.00 | 1.96 |  | 6.21 | 3.46 |
| *Phoma* | 0.87 | 0.07 |  | 1.66 | 0.11 |  | 1.95 | 0.09 |  | 4.42 | 0.13 |
| *Cladosporium* | 2.42 | 2.10 |  | 3.01 | 2.42 |  | 1.10 | 0.87 |  | 2.58 | 1.89 |
| *Alternaria* | 1.08 | 0.21 |  | 1.50 | 0.36 |  | 2.70 | 0.43 |  | 3.59 | 0.64 |
| *Corticiaceae_uc* | 2.19 | 0.11 |  | 1.71 | 0.08 |  | 1.67 | 0.10 |  | 1.28 | 0.05 |
| *Basidiomycota_uc* | 1.57 | 0.07 |  | 1.63 | 0.07 |  | 2.26 | 0.03 |  | 0.97 | 0.02 |
| *Penicillium* | 0.79 | 0.73 |  | 1.20 | 0.96 |  | 1.49 | 1.28 |  | 2.19 | 1.89 |
| *Physisporinus* | 0.45 | 0.00 |  | 1.61 | 0.04 |  | 0.25 | 0.00 |  | 1.54 | 0.01 |
| *Coriolaceae_uc* | 1.00 | 0.11 |  | 1.29 | 0.08 |  | 0.80 | 0.10 |  | 0.79 | 0.05 |
| *Acremonium* | 0.09 | 0.09 |  | 0.07 | 0.07 |  | 0.93 | 0.69 |  | 1.90 | 1.31 |
| *Clitocybe* | 0.16 | 0.16 |  | 1.29 | 1.25 |  | 0.02 | 0.03 |  | 1.48 | 1.48 |
| *Agaricomycetes_uc* | 0.78 | 0.62 |  | 0.95 | 0.65 |  | 0.61 | 0.47 |  | 0.76 | 0.41 |
| Uncultured | 0.00 | 23.79 |  | 0.00 | 18.54 |  | 0.00 | 32.19 |  | 0.00 | 22.04 |
| Others | 32.71 | 48.23 |  | 39.17 | 49.90 |  | 33.04 | 46.22 |  | 40.57 | 50.73 |
